# Three-dimensional Aerographite-GaN hybrid networks: Single step fabrication of porous and mechanically flexible materials for multifunctional applications

**DOI:** 10.1038/srep08839

**Published:** 2015-03-06

**Authors:** Arnim Schuchardt, Tudor Braniste, Yogendra K. Mishra, Mao Deng, Matthias Mecklenburg, Marion A. Stevens-Kalceff, Simion Raevschi, Karl Schulte, Lorenz Kienle, Rainer Adelung, Ion Tiginyanu

**Affiliations:** 1 Institute for Materials Science, Christian Albrechts University of Kiel, Kaiserstr. 2, D-24143 Kiel, Germany; 2 National Center for Materials Study and Testing, Technical University of Moldova, Chisinau, Republic of Moldova; 3 Department of Physics and Engineering, State University of Moldova, Chisinau, Republic of Moldova; 4 Institute of Electronic Engineering and Nanotechnologies, Academy of Sciences of Moldova, Chisinau, Republic of Moldova; 5 Institute of Polymers and Composites, Hamburg University of Technology, Denickestr, 15, D-21073 Hamburg, Germany; 6 School of Physics, University of New South Wales, NSW 2052 Sydney, Australia

## Abstract

Three dimensional (3D) elastic hybrid networks built from interconnected nano- and microstructure building units, in the form of semiconducting-carbonaceous materials, are potential candidates for advanced technological applications. However, fabrication of these 3D hybrid networks by simple and versatile methods is a challenging task due to the involvement of complex and multiple synthesis processes. In this paper, we demonstrate the growth of Aerographite-GaN 3D hybrid networks using ultralight and extremely porous carbon based Aerographite material as templates by a single step hydride vapor phase epitaxy process. The GaN nano- and microstructures grow on the surface of Aerographite tubes and follow the network architecture of the Aerographite template without agglomeration. The synthesized 3D networks are integrated with the properties from both, i.e., nanoscale GaN structures and Aerographite in the form of flexible and semiconducting composites which could be exploited as next generation materials for electronic, photonic, and sensors applications.

Nanoscale structures from different semiconductors have played an important role in the development of modern technological devices because of their exceptional physical and chemical properties^1^,^2^,^3^,^4^. Particularly nano- and microstructures based on GaN are extremely promising candidates for the next generation nanoelectronic, nanopiezotronic, and photonic devices^3^,^5^,^6^,^7^, light emitting diodes and lasers^8^,^9^,^10^, chemical and bio-sensors^11^. Recently reported phenomena like water splitting have also opened opportunities for their utilization for renewable energy resources^1^,^7^,^8^,^12^,^13^,^14^. Important features like direct and wide bandgap (~3.4 eV at room temperature), high mechanical stability, and large electrical/thermal conductivity promote GaN nano- and microstructures for numerous other challenging applications^15^,^16^,^17^. Being technologically a very important material, various synthesis techniques have been utilized for versatile growth of different GaN nano- and microstructures^18^,^19^,^20^,^21^,^22^. However, almost all of these structures are based on one or two dimensional epitaxially grown architecture, e.g., nanorods, nanowires, nanotubes etc. and their arrays on particular substrates^2^,^10^,^23^,^24^,^25^. In the case of epitaxial growth, the requirement of single crystalline substrates with fitting lattice parameters limits the choice and size of substrates and hence the production of large quantities of GaN nano- and microstructures. Although there exist several techniques which enable the growth of various GaN nano- and microstructures, their appropriate integration with externally associated devices is a highly challenging task when it comes to real applications^26^. Multistep processes, for example synthesis followed integration, have been applied to fabricated nanostructures based devices but this approach is very time consuming and equally expensive. In order to overcome these issues, either direct growth of semiconductors on the desired substrates/chips^27^,^28^ or the fabrication of large 3D interconnected networks made from these nano- and microstructures building blocks must be realized by appropriate techniques^29^,^30^,^31^. Such three dimensionally interconnected network structures exhibit all the features inherent to nanoscopic dimensions and in addition they are very easy to handle and hence they are even more flexible for various advanced applications. Indeed, there has been significant progress in growth of 3D interconnected networks from various semiconductors but for gallium nitride it is still an open issue. Here we report on a new method to synthesize GaN nano- and microstructures based 3D hybrid networks using highly porous microtubular Aerographite templates. Carbon based materials like graphene, carbon nanotubes, carbon nanorings etc. have already gained lot of research interest due to their fascinating and important properties suitable for various applications^32^,^33^. Particularly, in the last couple of years, the development of carbon based three-dimensional porous network materials like 3D graphene networks, have attracted intense attention because of their high technological importance^34^,^35^,^36^,^37^,^38^,^39^,^40^,^41^,^42^,^43^. Utilization of these porous 3D networks as templates can lead to non-agglomerated growth of nanostructures in the form of composite materials which is a very important application requirement^44^,^45^. The motivation behind the selection of a carbon based network as the template was to integrate the properties from both, carbon as well as GaN nano- and microstructures to synthesize a novel hybrid 3D network equipped with multifunctional properties. Different structures like graphene and graphene oxide etc., from the carbon family have already been utilized for the growth of GaN nano- and microstructures and they have also shown promising outcomes^46^,^47^,^48^,^49^,^50^,^51^,^52^ but their role has been mainly limited to templates. However, the use of carbon based networks as templates for growth of GaN nano- and microstructures in the form of 3D hybrid networks where the template exhibits double role, i.e., as a backbone and a functional component in the finally grown 3D network material, is very rarely reported in literature, to the best of our knowledge.

The template employed here is the recently introduced Aerographite (AG) material which is a super-light weight, extremely porous, mechanically flexible, graphite based 3D network built from interconnected struts of hollow graphite tubes with micron-scale diameters and a nanoscopic (~15 nm) wall thickness^53^. The Aerographite network is an appropriate template choice because it exhibits extraordinary properties suitable for different applications and also fulfills all the requirements for being a template material because of its high porosity, open pore structure, and mechanical stability features. In the present work, we demonstrate the direct growth of GaN nano- and microstructures on the surface of Aerographite tubes in the form of a AG-GaN 3D hybrid network (NW) using rapid hydride vapor phase epitaxy (HVPE) method. The synthesized 3D hybrid porous network material built from a network of Aerographite tubes decorated with GaN nano- and microstructures (abbreviated as AG-GaN) combines the fundamental properties of both GaN nanostructures and Aerographite. The morphological evolution of the grown GaN nano- and microstructures in the AG-GaN hybrid network was investigated by scanning electron microscopy (SEM). Detailed X-ray diffraction and high resolution transmission electron microscopy (HRTEM) investigations confirmed the crystalline nature of GaN structures. Electromechanical measurements on the AG-GaN 3D hybrid network demonstrated the mechanical flexibility and proved that the applied stress has strong influence on the electrical current output. The AG-GaN 3D hybrid network equipped with properties like low density, excellent electrical conductivity, mechanical flexibility, cathodoluminescence, etc. could be a very promising candidate for various potential applications.

## Results and Discussion

**Figure 1:**
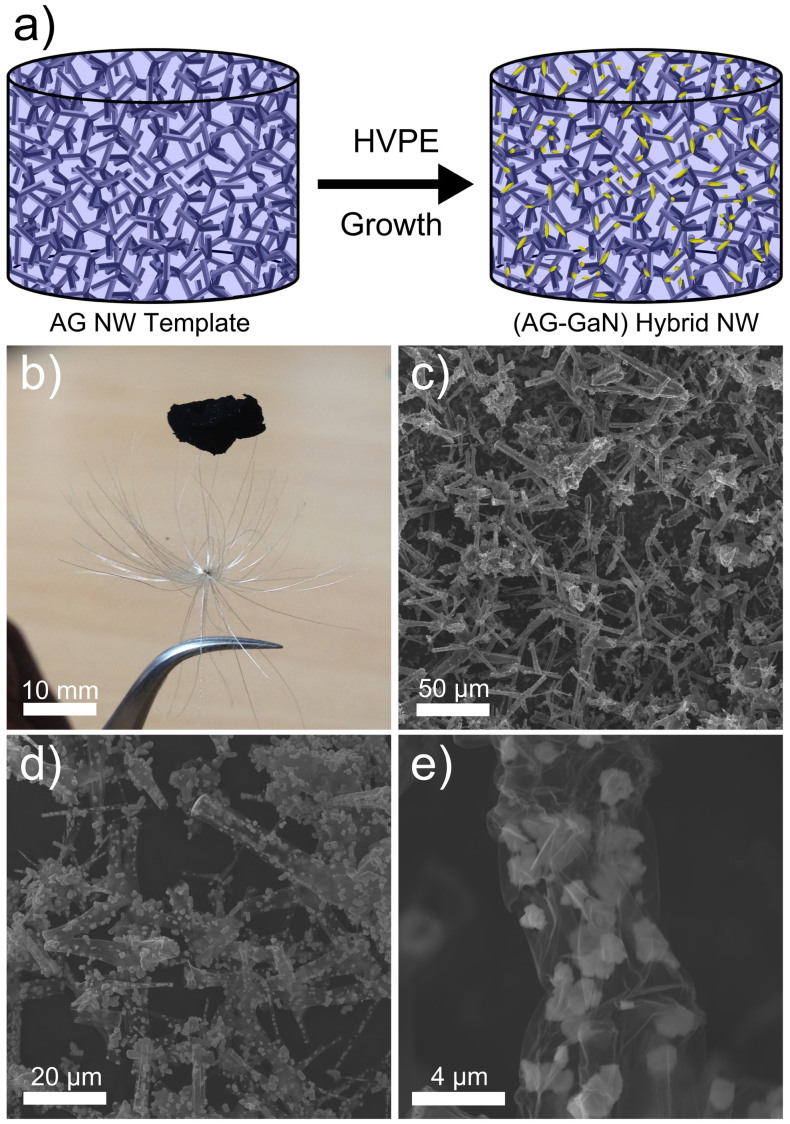
Synthesis of Aerographite-Gallium Nitride (AG-GaN) 3D hybrid network (NW) from Aerographite template. (a) Schematic representation for the growth of (AG-GaN) hybrid network on AG template in a single step HVPE growth process. (b) Digital photograph of AG-GaN hybrid network placed on a lightweight plant seed. (c–e) Low to high magnification scanning electron microscopy images from grown AG-GaN hybrid NW showing the growth of GaN nano- and microstructures on the tubular network of the AG template.

The growth principle of the AG-GaN hybrid 3D network from the Aerographite template is demonstrated by the scheme shown in Figure 1(a). The 3D AG network consisting of a microtubular network is mounted in the HVPE chamber which enables the growth of GaN nano- and microstructures on the surface of AG microtubes while retaining the 3D architecture of the AG template. Figure 1(b) corresponds to the digital camera image of the grown AG-GaN hybrid network which is almost black (similar to AG material). The colour of the hybrid network in general depends upon the areal coverage of GaN nano- and microstructures on the AG tubes and the 3D templates which are almost completely covered by GaN structures appear to be grayish in colour. Detailed SEM studies on the grown 3D AG-GaN network were performed and corresponding images (low to high magnification) are respectively shown in Figure 1(c–e). The overview SEM images (Figure 1c,d) taken from the hybrid network demonstrate that after HVPE process, the template architecture is maintained however a corresponding high magnification SEM image in Figure 1(e) confirms the growth of GaN nano- and microstructures on the surface of electron-transparent Aerographite microtubes.

**Figure 2:**
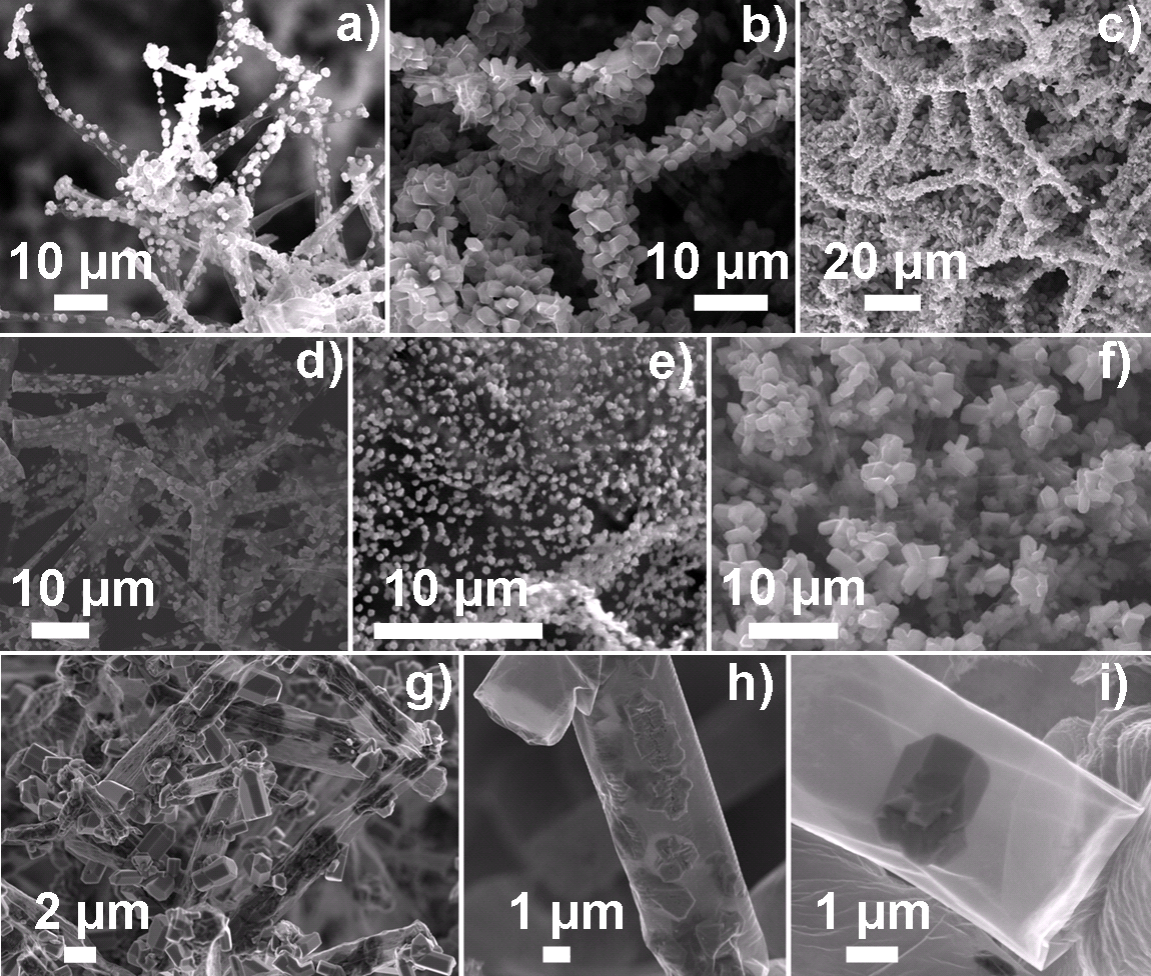
SEM micrographs of the Aerographite-GaN 3D hybrid network. (a–c) Growth of the GaN nano- and microcrystals on the Aerographite tabular network from partial to complete coverage. (d–f) Uniformity and morphology of the grown GaN nano- and microstructures on the AG tubes. (g) Illustrates the growth of GaN nano- and microcrystals on the both surfaces (inner and outer) of the Aerographite tubes. (h–i) Low and corresponding high magnification SEM images demonstrating the growth of hexagonally facetted GaN nanocrystals at the inner surface of the tube.

The micro-structural evolutions corresponding of the AG-GaN 3D hybrid network have been investigated in more detail by SEM and corresponding images are shown in Figure 2. During HVPE process, the GaN nano- and microstructures grow on the surface (both outer and inner one) of Aerographite tubes in the template. Despite of the fact that GaN nano- and microstructures are almost ~30000 times heavier (density ratio of GaN to AG) with respect to Aerographite, the 3D structural integrity of the AG template, which represents an interconnected network of hollow tetrapods, is maintained. Monitoring the experimental parameters, e.g., growth time in the HVPE process, offers the possibility of achieving the desired growth (arial coverage, shape morphology etc.) of GaN structures on the AG tubes in the 3D porous template. SEM images in Figures 2a–2c demonstrate the arial covering (from partial to full) of 3D AG microtubular network with GaN nano- and microstructures. A longer time HVPE process enables the growth of interconnected network of GaN nano- and microstructures on AG tubes (Figure 2c) and this is attributed to the fact that once the entire surface of AG tubes is covered, further growth can only occur over the already grown structures. It is very important to mention here that with increasing the amount of GaN nano- and microstructures on the AG tubes (from partial to full coverage, even up to their network), the architecture of 3D AG template remains intact as revealed by Figure 2c. For the growth of such hybrid porous materials from light weight constructions, the initial template architecture is quite important and for better visualization, SEM images corresponding to the pure AG network (used as template here for GaN growth) are also shown in the Supplementary information, see Figure S1. The macroscopic (volume ~1 cm^3^) 3D template network (AG) is entirely made from interconnected tetrapod building blocks with Aerographite tubes as arms (Figure S1). The tetrapod shape appearance and structural integrity of the Aerographite network is a direct consequence of the initial sacrificial ZnO 3D template in which microscale ZnO tetrapods form an interconnected network (Figure S2a). These Aerographite networks were synthesized by the direct conversion of appropriate ZnO 3D templates in chemical vapor deposition process in a single step^53^. This method also offers the possibility to interrupt the Aerographite synthesis process at the intermediate stages and therefore 3D templates in ZnO/Aerographite composite form (SEM images in Supplementary information, Figure S2b–2d) can be utilized if required for certain applications^47^. Recent reports on growth and properties of GaN based hybrid materials utilizing carbon nanotubes/graphene, ZnO etc.^54^,^55^,^56^ show the high perspectives of such AG-GaN hybrid 3D networks in future applications. However, in the present case for the GaN growth, pure AG networks have been utilized as templates in HVPE process. Unlike solid arms of ZnO tetrapods (the sacrificial template for AG synthesis), the structures in the pure Aerographite network are hollow graphitic tubes with micrometer-scale diameters and wall thickness in the nanoscopic region on which the growth of GaN nano- and microstructures has occurred in the HVPE process.

**Figure 3:**
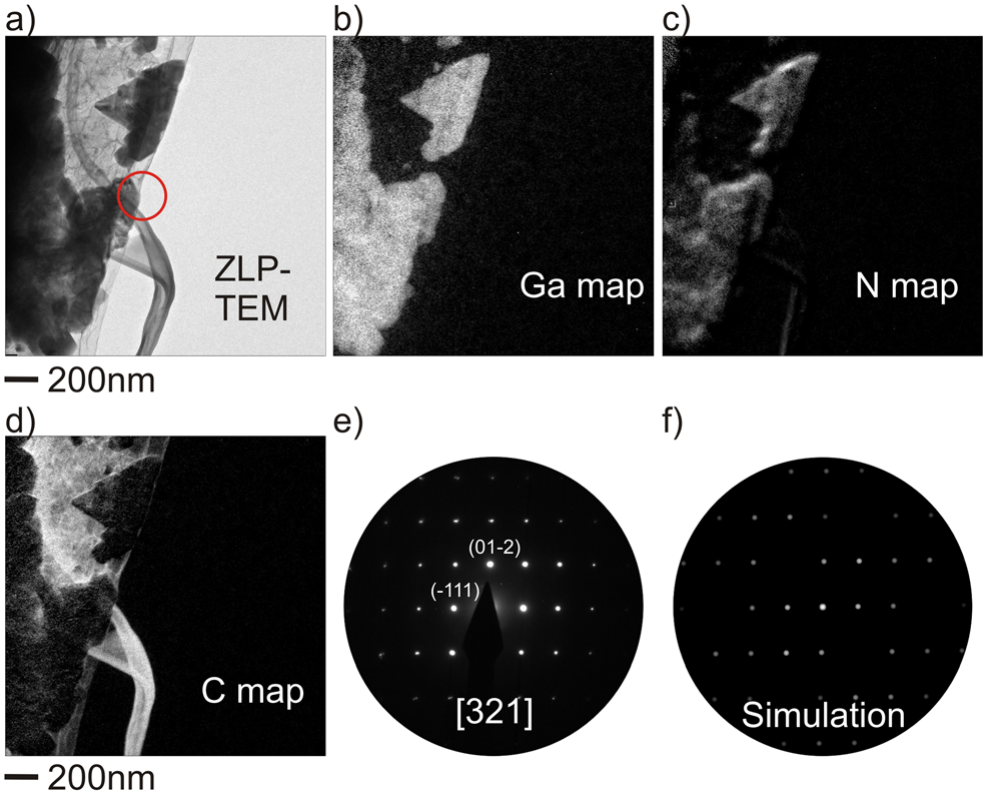
EFTEM and SAED investigations on AG-GaN 3D hybrid network. (a) Zero loss peak TEM image of a tubular Aerographite arm partially coated with GaN nano- and microstructures. (b)–(d) Corresponding EFTEM maps of gallium, nitrogen, and carbon indicating the formation of GaN onto AG. (e) SAED pattern along the zone axis [321] of GaN recorded from the circled area in the ZLP-TEM image. (f) Kinematic simulation of the [321] axis pattern.

Growth of GaN nano- and microstructures over the AG tubes exhibits a very interesting feature in the sense that there is no need of epitaxial substrates, however, controlling the morphology and uniformity of the GaN structures grown on AG tubes is an equally important and highly desirable requirement. In the present case of GaN growth on AG tubes using HVPE process, it is possible to control the uniformity and morphology (e.g. by varying experimental parameters such as deposition time and growth temperature) of GaN nano- and microstructures in the AG network as shown by SEM images, see Figure 2d–2f. Uniform growth of well separated/nearly agglomerated GaN nanostructures on AG tetrapods is clearly visible in SEM, Figures 2(d) and 2(e) respectively, however, SEM image in Figure 3(f) demonstrates the agglomerated growth (overgrowth conditions) of hexagonal faceted GaN nano- and microcrystals. In order to get further insights about the growth behaviour, detailed SEM studies were performed on a hybrid specimen exhibiting rather low density of GaN nano- and microcrystals grown on AG, and corresponding images are shown in Figure 2(g)–2(i). From the SEM images it is very clear that GaN growth does not occur only on the outer surface (Figure 2b, 2c, 2f, 2g), but also on the inner surface (Figures 2h–2i) of the AG tubes and this particular growth behaviour is mainly due to fascinating morphology of AG tubes in the 3D templates as described in the following section.

High resolution SEM studies also revealed that the walls of individual hollow graphitic tubes are in general non-porous, however, they do exhibit some openings or pores especially at the junctions and the tips^53^. Since the growth of GaN nanostructures is initiated via vapor transport in the HVPE process, in principle the formation of a continuous layer of GaN films entirely around the graphitic tubes is expected, however random growth of individual GaN nano- and microstructures has been observed. Figure 2h demonstrates the clear growth of GaN structures at the inner surface of the tube and this most likely occurs due to the penetration of the HVPE reactants into the AG tubes of the network through holes/pores in the graphitic structure. This is also a confirmation of the long diffusion length of the reactants inside AG tubes. An example of a hexagonal-prism like GaN nano- and microstructure grown at the inner surface of the graphitic microtube is illustrated by SEM image in Figure 2i. To further illustrate the shape evolution of GaN crystals on AG tubes, a high resolution SEM image from AG-GaN specimen is shown in Figure S5 (Supplementary information) which demonstrates the growth at outer and inner surfaces of the AG tube with focus on the shape of the GaN crystal at the outer surface of AG tube. It has to be noted that in most cases the GaN structures exhibit a hexagonal prism-like shape with different orientations which clearly indicate the free growth of the GaN on AG surface. Thus, growth of GaN nano- and microstructures takes place on both the inner and the outer surfaces of the graphitic carbon microtubes which are constituent elements of the Aerographite network. The obtained AG-GaN hybrid network material is also mechanically flexible which allows one to reduce its volume easily by compression, thus resulting in a controlled variation of the density and surface area per volume of GaN nano- and microstructures. It is a very important aspect that the specific 3D architecture of the AG network is unaffected by the deposition of GaN nano- and microstructures (Figures 1d, 2a–2d, Figure S4). Figure S4 shows a comparison of a periphery area of pristine Aerographite with a similar area after the deposition of GaN nano- and microstructures. The graphitic microtube architecture could even sustain high loading densities of GaN nano- and microstructures on the surfaces without structural deformation, see Figure 2b (Figure S4b–d), which indicates the applicability of Aerographite templates towards fabrication of new 3D hybrid materials. The mechanical flexibility, unique surface morphology, and direct growth possibility etc. enable these highly porous Aerographite networks to be appropriate backbones for non-agglomerated growth of active nanostructures (e.g., GaN in present work) and therefore of multifunctional 3D composites.

The crystalline nature of the HVPE deposited GaN nano- and microstructures in the 3D hybrid network were studied by detailed XRD measurements (~10 hours scan). The obtained Bragg reflections {(100), (002), (101), (102), (110), (103), (200), (112), (201), (004)} in the XRD pattern (Figure S6a) inherently belong to GaN^57^. The XRD diffractogram is almost similar to that of a randomly dispersed GaN crystals^58^ which is also in close agreement with the obtained GaN nano- and microstructures with different crystallographic orientations in the AG-GaN hybrid networks. XRD diffractogram does not exhibit any visible reflections related to graphitic carbon (expected 2θ value ~42°, 44°, and 54°) which is very likely due to the detection limit of the XRD set-up under applied 10 hours scan conditions. The fabrication of the AG-GaN hybrid network involves several processing steps, i.e., conversion of porous ZnO templates into Aerographite networks inside a CVD chamber followed by growth of GaN nano- and microstructures on AG network in HVPE process. Therefore it was very important to investigate which type of elemental species does exist in the AG-GaN network specimen and for this detailed EDX investigations using SEM (elemental mapping) and high-resolution-TEM (including energy filter) have been performed (Supplementary Information, Figure S5b–d). The EDX results confirmed the presence of C, N, Cu, and Ga elements in the hybrid network and the absence of Zn. The observed C content in the EDX spectrum (Figure S6b) mainly originated from AG tubes, but a small contribution of the carbon coating from the used TEM-grid must also be considered. Cu occurred due to the use of a copper TEM grid during TEM measurements. However, no signature of Cu has been observed in EDX measurements inside the SEM (Figure S6c–d).

Detailed TEM investigations on the HVPE synthesized AG-GaN specimen are demonstrated in Figure 3. The chemical composition has been examined by energy filtered TEM (EFTEM) elemental mapping and the crystal structure of the grown GaN nanostructures was analyzed by selected area electron diffraction (SAED) studies. Figure 3a shows a zero loss peak (ZLP)-TEM micrograph of the region of interest. The marked region with a circle indicates the location where SAED has been recorded. Figure 3(b–d) shows the energy filtered TEM elemental maps of a graphitic tube partially covered with GaN nano- and microstructures. The EFTEM maps also confirmed the presence of Ga and N within the structures. The SAED pattern (Figure 3e) has been indexed assuming the structure of GaN (zone axis [321] space group: P6_3_*mc*) and Figure 3f shows the corresponding simulated pattern for the zone axis [321]. Several reflections {e.g. indices: (-12-1), (-333), (11-5)} are strongly excited by dynamical scattering effects which can be attributed to the thickness of the bulky GaN crystals. The comprehensive results of structure and elemental investigations by the TEM proved a successful synthesis of crystalline GaN nano- and microstructures on Aerographite (Figure S7).

**Figure 4:**
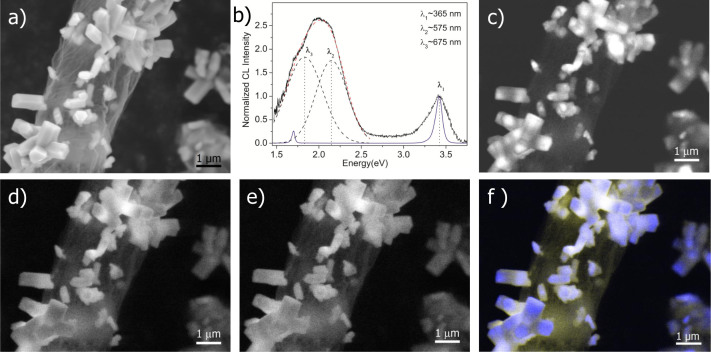
Cathodoluminescence from the HVPE synthesized AG-GaN 3D hybrid network: (a) SEM image taken from a fragment of AG-GaN hybrid network, (b) CL spectrum corresponding to AG-GaN specimen in (a), Monochromatic micro-CL images for (c) Ultra-violet, (d) Yellow, (e) Red emissions respectively. (f) UV-yellow color composite micro-CL image corresponding to AG-GaN specimen in (a). [The violet curve in figure 4(b) corresponds to the CL spectrum of bulk crystal GaN in which the CL peak at ~3.4 eV is attributed to GaN and the ~1.7 eV peak is the corresponding second order diffraction artifact from the grating].

Since GaN is an important material for optical applications, detailed cathodoluminescence (CL) studies of AG-GaN hybrid network specimen have been performed and corresponding results are demonstrated in Figure 4. It is observed that the GaN nano- and microstructures exhibit two rather intense luminescence bands, the near edge UV emission peak with the maximum intensity at ~365 nm (~3.4 eV) and a broad multicomponent defect peak associated emission at ~2 eV (Figure 4b). This broad emission band consists of the yellow luminescence (YL)^59^,^60^,^61^,^62^ with maximum intensity at ~575 nm (~2.2 eV), usually attributed to host lattice defects, and a red luminescence with maximum intensity at ~675 nm (~1.8 eV). For reference, a typical CL spectrum (see solid blue line) corresponding to bulk GaN crystal is also shown in Figure 4(b) which exhibits two peaks, i.e., ~3.4 eV and 1.7 eV. The CL peak ~3.4 eV in the reference spectrum corresponds only to bulk GaN crystal^63^ and the other peak at ~1.7 eV is the second order diffraction artifact from the grating. Figure 4a illustrates a SEM image taken from an area of AG-GaN hybrid network with GaN nano- and microstructures grown on the inner and outer surfaces of the graphitic carbon microtubes, while Figure 4b shows the corresponding CL spectrum recorded at the same position. Figure 4 (c–e) demonstrate the monochromatic micro-CL images for UV, yellow and red emissions, respectively. The UV-yellow color composite micro-CL image is presented in Figure 4f. It is observed that both the spectral distribution and the intensity of the luminescence vary along the length of nano- and microstructures. This feature, inherent to GaN nano- and microcrystals grown by HVPE^64^, is attributed to non-uniform distribution of impurities and host defects in the crystalline matrix.

**Figure 5:**
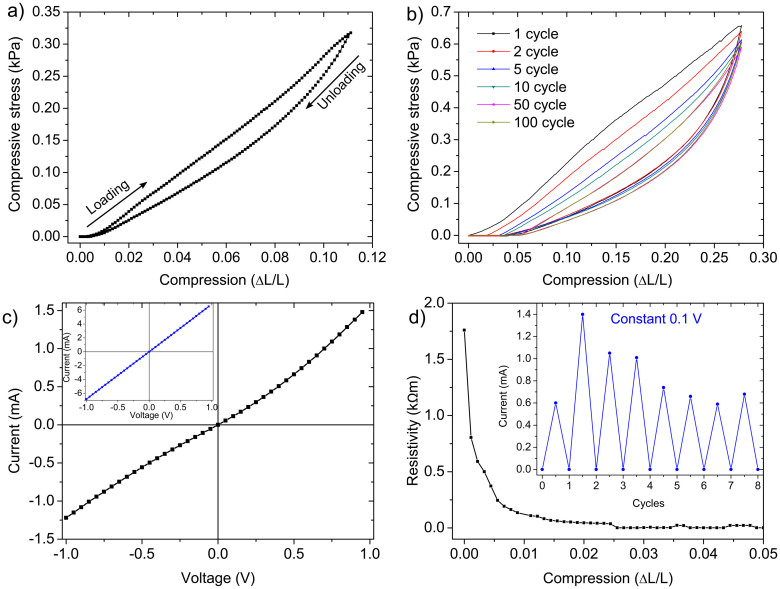
Electromechanical investigations on Aerographite-GaN nano- and microstructures 3D hybrid network. Cyclic loading-unloading response (compressive) of the AG-GaN network under compressive stress: (a) Single cycle, (b) multiple cycles (data up to 100 cycles is shown). (c) Current-voltage response of AG-GaN network showing non-Ohmic behaviour of current. The inset in (c) corresponds to I-V behaviour of pure Aerographite network which is showing Ohmic nature. (d) Shows the decrease in resistivity under compressive strain. The inset in (d) demonstrates the change in current values (extremes) under loading and un-loading cycles (current values have been extracted from the cyclic loading-unloading data shown in (b)).

For any three dimensional network structures made from interconnecting nanoscopic building blocks, mechanical stability is one of the first and foremost desired aspect with regard to their appropriate utilization. Detailed electromechanical studies have been performed on the synthesized AG-GaN 3D hybrid network and corresponding results are demonstrated in Figure 5. A typical stress (compressive) - strain response (single cycle) of the 3D hybrid network under cyclic loading and unloading is shown in Figure 5a which reveals that the network is very soft and mechanically flexible with rubber like elastic modulus behaviour. Figure 5b shows the stress-strain behaviour of the hybrid network corresponding to 100 loading-unloading cycles. It can be observed that after some cycles, the network exhibits a plastic deformation which is very obvious from the hierarchical tubular structure of the AG template used for growing the hybrid network. The feature of electrical conductivity of any nano- and microstructure directly enables it for various applications and flexible 3D network structures are even more promising because they can be directly integrated in the appropriate devices in any desired form. The current-voltage (I–V) behaviour of the synthesized AG-GaN hybrid network is presented in (Figure 5c) and compared with that of pure Aerographite (inset in Figure 5c). In contrast to pure Aerographite which shows Ohmic behaviour, the hybrid network exhibits a slightly non-linear I–V response, confirming the integration of the GaN nano- and microstructures into the hybrid network. The observed non-linear I–V characteristic could be attributed to the formation of non-Ohmic contact points between the GaN structures and Aerographite. The contribution from GaN nano- and microstructures to the electrical conductivity of the hybrid network mainly depends upon their density, distribution, etc. SEM images in Figure 1 and Figure 2 revealed the growth of GaN nano- and microstructures on both outer and inner surfaces of the microtubular Aerographite network. Since the hybrid network is flexible, the stress (compressive) dependent resistivity behaviour has been measured and is shown in Figure 5d. Under compression, the resistivity of the AG-GaN 3D hybrid network is decreased and this is mainly due to increase in resultant number of electrical contacts. After removal of stress the original value of the resistivity is again achieved. Thus, the mechanical flexibility of the hybrid network leads to cyclic variations in electrical current as shown by inset in Figure 5d (data extracted from cyclic loading and unloading experiments in Figure 5b). This makes it possible to monitor the internal damage in the network by monitoring the electrical current. The stress dependent electrical conductivity of the synthesized hybrid network could be utilized in several applications like pressure sensors, actuators, self-reporting materials etc.

The growth of GaN nano- and microstructures on the surface of a microtubular Aerographite network has been directly (without any epitaxy requirements) realized in a single step HVPE process and based on structural/microstructural results, the possible growth mechanism involved is discussed here. The SEM images as well as the XRD measurements demonstrate that there is no preferential growth direction, e.g., c-texture, of the GaN nano- and microstructures onto and inside the Aerographite network. This indicates a direct growth of the GaN structures and excludes any kind of highly oriented growth like in epitaxy. As already mentioned, one can speculate that during the very early nucleation stages the GaN nanocrystals preferentially grow at the locations where the graphitic structure is disturbed by sp^3^ hybridized carbon. From electron energy loss spectroscopy studies (EELS) at Aerographite, it is observed that the microtubes contain sp^3^ hybridized carbon which is indicative of a disturbed crystal structure of the graphitic walls^53^. Apart from sp^3^ bonded carbon atoms, the Aerographite tubes also exhibit other surface defects like kinks or terraces (adopted from the ZnO sacrificial template during conversion) which all together contribute to the crystallization process of GaN structures on the surface of Aerographite tubes. The long diffusion length of the reactants along the surface of the graphitic tubes allows the reactants to migrate until they participate in the nucleation and growth process. With proceeding growth, additional GaN nanostructures start to grow adjacent to previously grown crystals. It is also very important to take once more into account the presence of GaN nano- and microstructures on the inner surfaces of AG microtubes after the HVPE process. The growth of GaN on the inner surface of the AG tubes can be explained by openings and pores in the Aerographite network which allow the HVPE reactants to enter into the hollow region of microtubes in the network. Once these reactants are inside the tubes, the seamless network allows the reactants to move almost freely over long distances until they are consumed in the growth of the GaN nano- and microstructures. With a high probability, the gas entry of the HVPE reactants occurs at the same openings which are responsible for the gas exchange of H_2_, H_2_O, and Zn during the AG synthesis. However, the observations showed that the surface density of the grown GaN nano- and microstructures, especially on the inner surface, depends on the type of AG template employed. This can be hypothesized due to the density of defects on the inner surface of the graphitic tubes which again depends on the ZnO template utilized and its temperature treatment during AG synthesis by CVD. One example for this surface dependency on the ZnO template is the corrugated type of Aerographite tubes which adopt their surface structure from a corrugated ZnO template^53^. The fabrication of AG-GaN 3D hybrid networks mainly depends upon the initial architecture of the AG template and growth parameters in the HVPE process. The AG template architecture can be easily tailored by the use of different ZnO templates during Aerographite synthesis (by CVD) and the HVPE process offers a controlled deposition of GaN nano- and microstructures on the AG template. Since this method offers a possibility to control both parameters, i.e., AG template architecture as well as GaN deposition, the AG-GaN 3D hybrid networks with desired features, like size, shape, density of GaN nano- and microstructures etc. can be easily synthesized for advance photonics^65^ and biomedical^66^ applications. Our fabrication strategy for three dimensional networks is very flexible as these hybrid networks can be further and easily loaded with different nanostructures for desired multifunctionality.

## Conclusions

In conclusion, direct and rapid growth of GaN nano- and microstructures on the surface of Aerographite tubes in the form of a 3D Aerographite-GaN hybrid flexible interconnected network by HVPE technique is demonstrated. In the HVPE process, homogeneous growth of GaN nano- and microstructures occurs on both, the inner and the outer surfaces of the graphitic tubes in the entire Aerographite network while several growth directions are indicative of the direct free growth. The grown GaN nano- and microstructures are found to be strongly attached on the surface of the thin graphite walls which prevent their agglomeration. Microstructural studies revealed the highly crystalline nature of these HVPE grown GaN nano- and microstructures in the hybrid network. The cathodoluminescence results showed that the GaN nano- and microstructures exhibit intense near-band-edge UV emission, and yellow emission usually attributed to host defects. The achieved AG-GaN hybrid 3D network retains the highly flexible behavior of the pure Aerographite template which opens up a wide range of application opportunities. In particular, the stress dependent electrical conductivity of the Aerographite-GaN hybrid 3D network can be useful for designing different kinds of sensors and self-reporting materials. The fascinating three-dimensional hybrid flexible network of the spatially distributed GaN nano- and microstructures on Aerographite may be exploited as next generation light-weight conducting composite materials for electronic, photonic, and sensors applications.

## Methods

### Synthesis of Aerographite 3D Templates

The here used Aerographite networks are produced by the one step chemical vapor deposition (CVD) process described with all parameters in our previous paper^53^. Highly porous ZnO networks with a 3D architecture, which are entirely built up from interconnected micrometer thick rods, often in the shape of tetrapods and multipods, are used as sacrificial templates^30^. These ZnO templates are converted by a (CVD) process which incorporates a heat treatment at 760°C in an Argon/Hydrogen atmosphere. Evaporated toluene provides carbon for the nucleation and growth of enclosing graphitic shells, whilst the underlying ZnO network is reduced and removed by the gas flow. Since the conversion process exactly follows the template structure, Aerographite material is almost exact mimicry from sacrificial ZnO template architecture in which ZnO has been replaced by carbon in the form of tubular graphitic carbon^53^.

### Growth of Aerographite-GaN 3D Networks

The as grown Aerographite networks (pure) have been used as templates for the growth of GaN nano- and microstructures in a hydride vapor phase epitaxy (HVPE) system equipped with four-temperature-zone-heated horizontal reactor. Metallic gallium, ammonia (NH_3_) gas, hydrogen chloride (HCl) gas, and hydrogen (H_2_) were used as source materials and carrier gases. In the source zone, GaCl was formed as a result of chemical reactions between gaseous HCl and liquid Ga at 850°C. The GaCl and NH_3_ gas reacted with each other in the react zone, where at the beginning the temperature was kept at 600°C for 10 min to initiate nucleation of GaN on the surface of graphite, and then increased up to 950°C for durations up to 10 min to produce GaN nano- and microstructures. In the process of GaN growth, the HCl, NH_3_, and H_2_ flow rates were equal to 15 sml/min, 500 sml/min and 3600 sml/min, respectively.

### Characterizations

The microstructural evolutions of pure-Aerographite and GaN nano- and microstructures hybrid networks were investigated by scanning electron microscopy instruments Zeiss Ultra Plus and VEGA TESCAN TS 5130MM. The compositional analysis of AG-GaN networks was carried out using EDX, in combination with SEM. X-ray diffraction studies were performed by using 3000 PTS Seifert machine (with 40 kV and 40 mA, Cu K_α_ radiation with *λ* = 1.541 Å). Transmission electron microscopy analysis was performed on a Tecnai F30 STwin electron microscope (300 kV, field-emission gun, spherical aberration constant Cs = 1.2 mm). Energy-filtered TEM (EFTEM) with a post-column Gatan Image Filter was used to obtain elemental maps of the sample. The TEM EDX analysis was performed in TEM mode with a Si/Li detector (EDAX System). 

A JEOL 7001F Field Emission SEM equipped with a Gatan XiCLone CL microanalysis system was used for comparative morphological and CL characterization. The monochromatic CL images were collected using a Peltier cooled Hamamatsu R943-02 High Sensitivity Photomultiplier Tube. The CL spectra and images were generated using 10 keV, 0.65–1.65 nA electron beam from 350 nm diameter areas of typical regions of the specimen. The spectra have been collected with a Princeton Instruments Pixis 100 UV optimized CCD.

### Electromechanical Measurements

The electro-mechanical measurements were conducted with a self designed/built computer controlled setup which consists of a Kern PLE 310-3N precision balance and a Märzhäuser Wetzlar HS 6-3 micromanipulator. The setup allows a stepwise tensile or compressive deformation of the sample up to an arbitrary number of cycles while the force is measured by the balance. The setup also allows the simultaneous measurement of the electrical resistance of the sample for each deformation step by a Keithley 2400 source meter which is connected to the sample by gold plated copper contacts.

## Author Contributions

A.S., Y.K.M., M.M., R.A. and I.T. designed the experiment. A.S., M.M., K.S. and R.A. synthesized the Aerographite templates. A.S., Y.K.M., T.B., M.A.S.K., S.R., R.A. and I.T. have grown the hybrid 3D networks, characterized and analyzed the data. M.D. and L.K. performed TEM investigations. A.S., Y.K.M., R.A. and I.T. wrote the paper.

## Supplementary Material

Supplementary Information Supplementary information
