# Supplementary material for: Three-dimensional Aerographite-GaN hybrid networks: Single step fabrication of porous and mechanically flexible materials for multifunctional applications
Source: Sci Rep. 2015 Mar 6;5:8839. doi: 10.1038/srep08839 (PMC4351516; doi:10.1038/srep08839)
Supplement: Supplementary Information [file srep08839-s1.doc]

Supplementary Information for

**Three-dimensional Aerographite-GaN hybrid networks: Single step fabrication of porous and mechanically flexible materials for multifunctional applications**

Arnim Schuchardt,^1^ Tudor Braniste,^2^ Yogendra K. Mishra,^1*^ Mao Deng,^1^ Matthias Mecklenburg,^3^ Marion A. Stevens-Kalceff,^4^ Simion Raevschi,^2^ Karl Schulte,^3^ Lorenz Kienle,^1^ Rainer Adelung,^1*^ Ion Tiginyanu^2*^

^1^Institute for Materials Science, Christian-Albrechts University of Kiel, Kaiser Str. 2, D-24143 Kiel, Germany

^2^National Center for Materials Study and Testing, Technical University of Moldova, State University of Moldova and Institute of Electronic Engineering and Nanotechnologies, Academy of Sciences of Moldova, Stefan cel Mare av. 1, MD-2001 Chisinau, Republic of Moldova

^3^Institute of Polymers and Composites, Hamburg University of Technology, Denickestr. 15, D-21073 Hamburg, Germany

^4^School of Physics, University of New South Wales, NSW 2052 Sydney, Australia

**Corresponding Authors:**

**Dr. Yogendra Kumar Mishra (**ykm@tf.uni-kiel.de**)**

**Prof. Dr. Rainer Adelung (**ra@tf.uni-kiel.de**)**

**Prof. Dr. Ion Tiginyanu (**tiginyanu@asm.md**)**

**Figure S1**

**
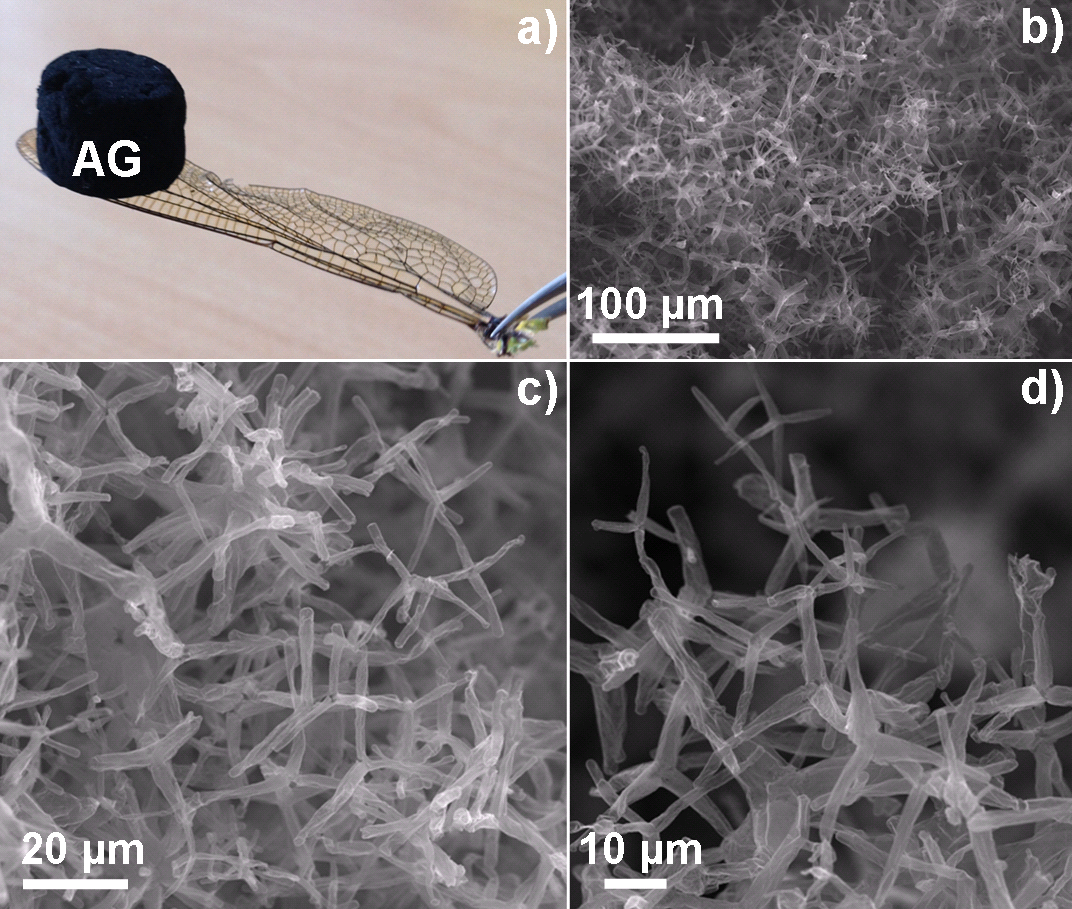
**

**Figure S1:** Morphology of pure Aerographite 3D network template. (a) Digital camera image of Aerographite (AG) network tablet. (b-d) Low to high magnification SEM images from AG network (a). The relatively high magnification images in (c) and (d) demonstrate that the 3D AG template utilized for GaN nano- and microstructures growth is pure (free from ZnO) and the network architecture is same as that of initial template material (Figure S2a, ZnO networks were used as sacrificial templates growth of Aerographite).

**Figure S2**

**
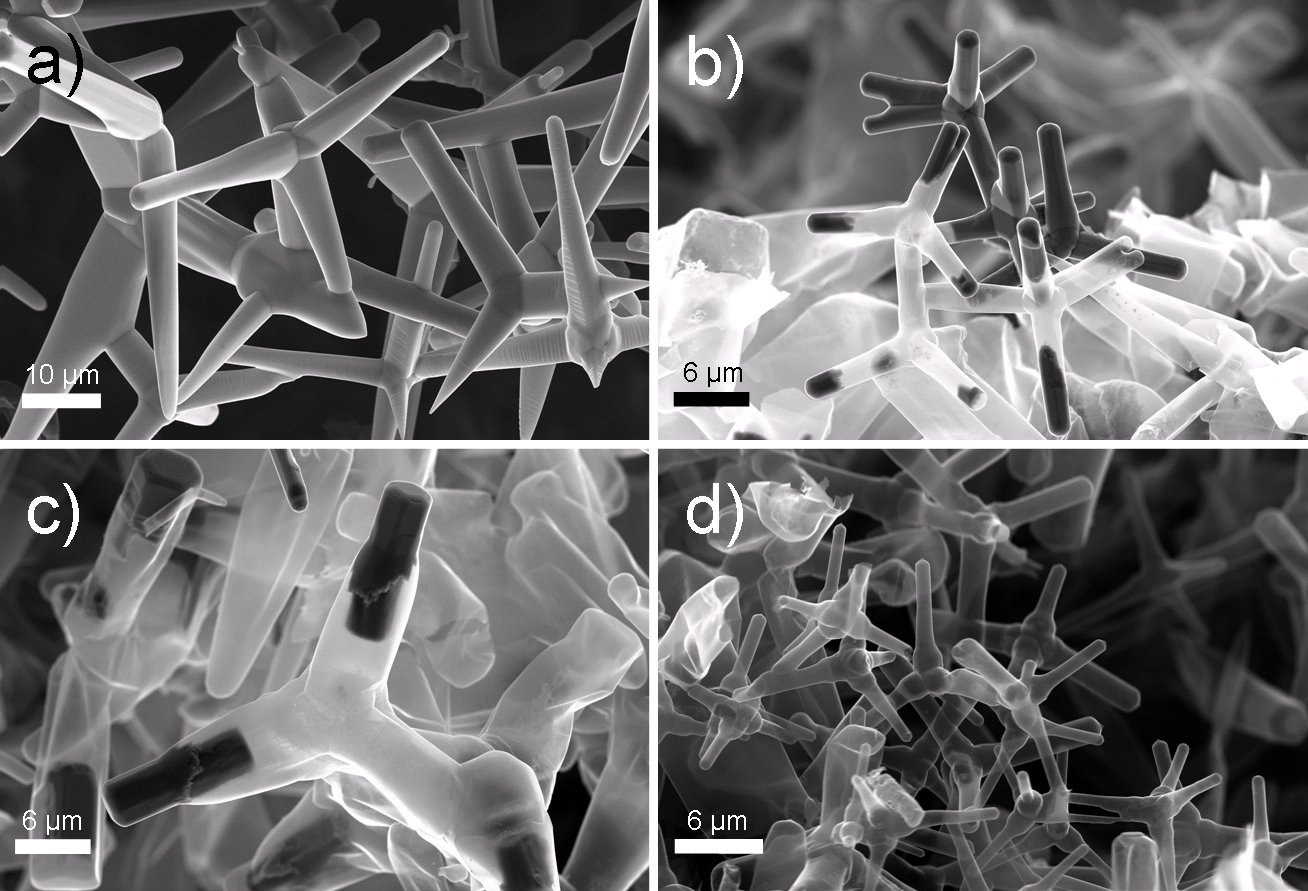
**

**Figure S2:** Overview of the different synthesis steps involved in the AG fabrication. **(**a) Free standing 3D ZnO network which is employed in the Aerographite synthesis as sacrificial template material. (b) Semi-converted AG network with ZnO residuals located at the tips of the Aerographite tetrapods. (c) Magnified view on a single semi-converted AG tetrapod achieved by an interrupted synthesis. (d) Aerographite specimen after the complete removal of the ZnO network template.

Figure S2 gives an overview of the different conversion states during the Aerographite synthesis. The sacrificial ZnO template network is shown in figure S2a. This image nicely illustrates the network junctions in between the ZnO tetrapods which have been formed during the post annealing of the ZnO networks. For the case of an interrupted synthesis, ZnO will remain inside of the graphitic tubes due to the insufficient hydrogen supply which is necessary to remove the ZnO (see figure S2b/S2c). This means that it is also possible to combine AG with GaN and in addition with ZnO. A typical example of completely converted Aerographite sample without any residual ZnO is shown in figure S2d. For the growth of GaN nano- and microstructures only the completely converted AG networks (Figure S2d) have been used as template materials in HVPE process.

**Figure S3**

**
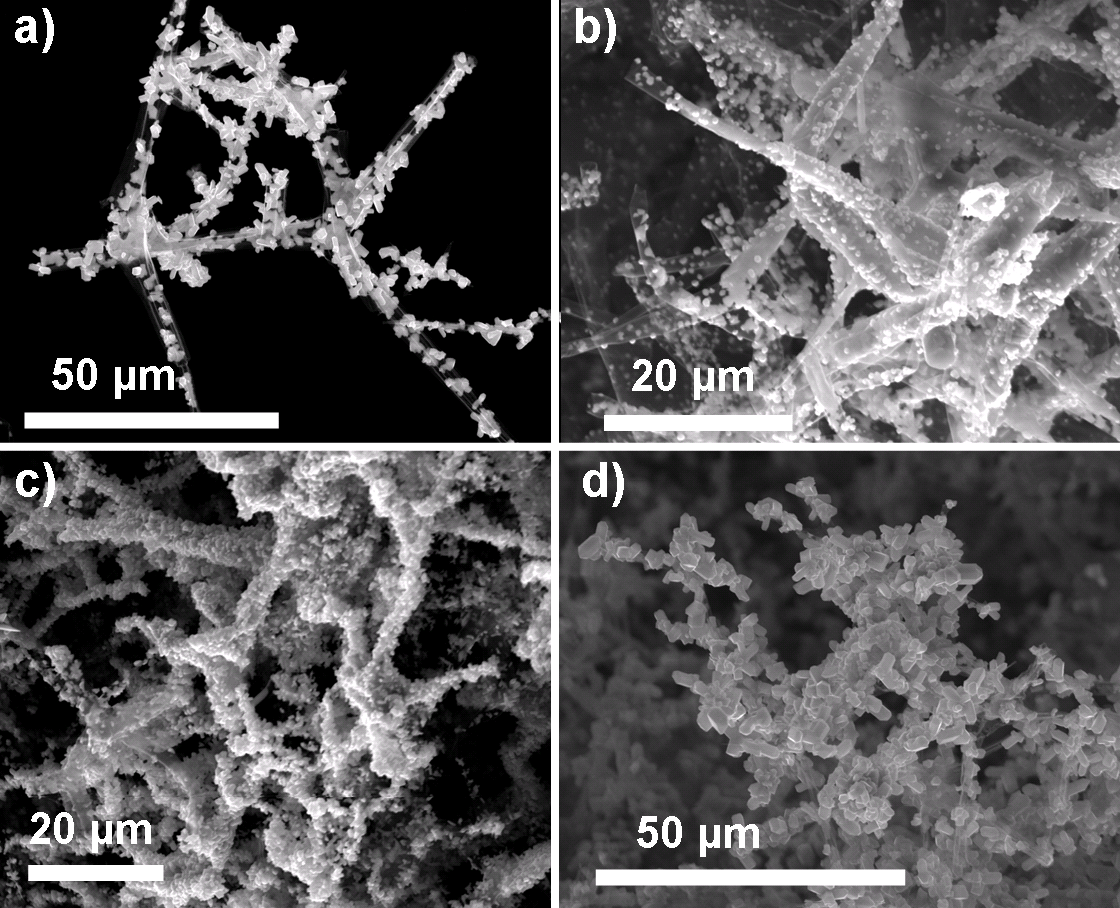
**

**Figure S3: (a-d)** SEM images of Aerographite tubular network loaded with increasing amounts of GaN nano- and microcrystals. (a) A large freestanding AG-GaN network showing that Aerographite tubes are strong enough to hold GaN nano- and microparticles on it. (b) AG-GaN hybrid network almost uniformly decorated with GaN nanocrystals. (c,d) AG-GaN hybrid 3D network equipped with large amounts of GaN nano- and microcrystals. SEM image in Figure S3d corresponds to AG microtubes with highly loaded GaN nano- and microstructures and it can be observed that even after such a high loading of GaN nano- and microstructures, the original architecture of AG network is maintained. This indicates that Aerographite can bear the upcoming stresses while it is filled decorated with GaN during the HVPE process.

**Figure S4**


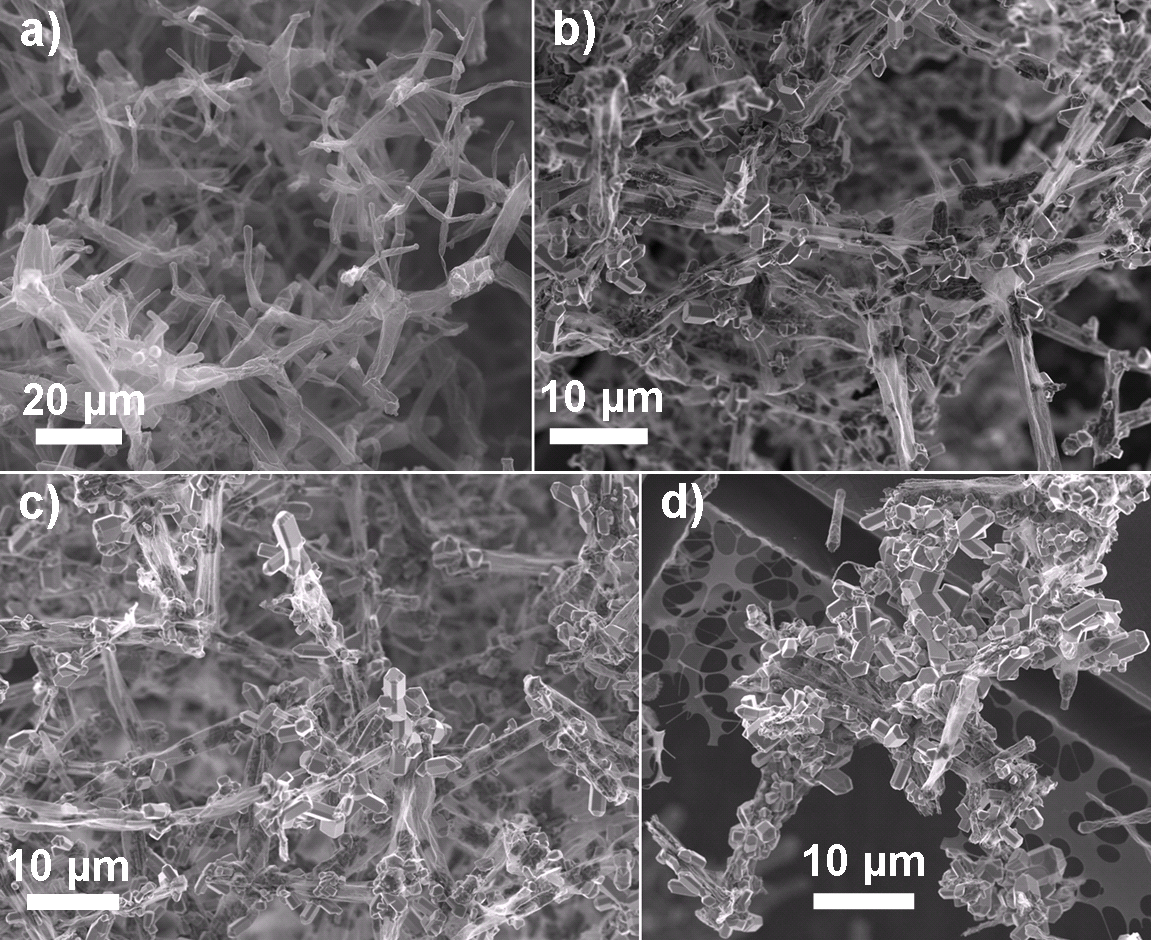


**Figure S4:** (a) As grown (pure) Aerographite network which has been used as template for GaN deposition in HVPE process. (b)-(d) Representative SEM images of AG microtubes loaded with hexagonally facetted GaN nano- and microstructures on them. (d) The image shows that large amount of hexagonal GaN nano- and microstructures can be deposited on AG tubular networks without destroying the original AG network architecture.

In Fig. S4a, an example of the pure Aerographite network (untreated) is shown. One can identify the tetrapodal architecture of AG which is genetically related to the sacrificial ZnO template. The AG network exhibits an open and a highly porous structure which can carry significant loads. The open and highly porous structure enables the reactants of used in the HVPE to penetrate deeply in the bulk of the Aerographite network. Figures S4b-d show SEM images of AG microtubes loaded with hexagonal GaN nano- and microstructures. Even at large amounts of loading the AG tubular networks with GaN nano- and microstructures, the template architecture does not collapse.

**Figure S5**

**
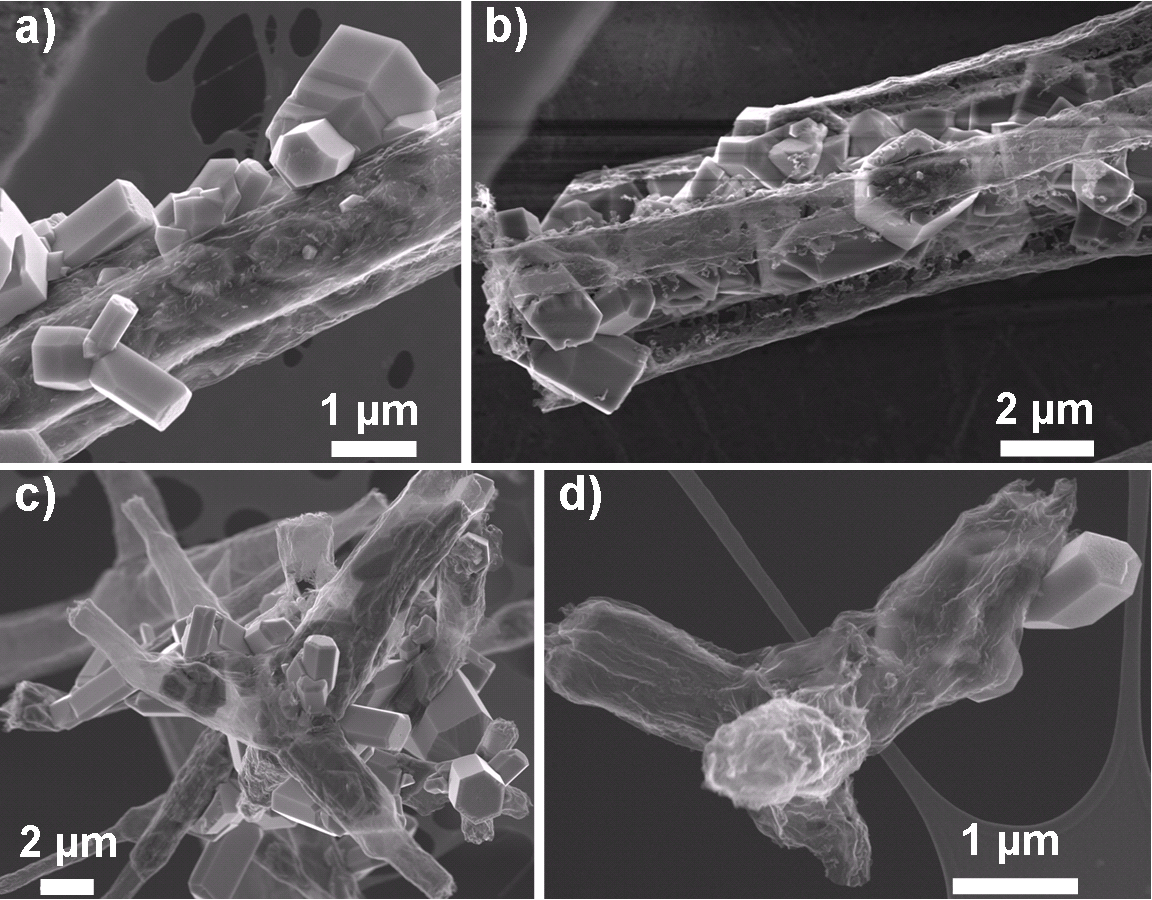
**

**Figure S5:** (a-d) High magnification SEM images corresponding to AG-GaN hybrid network specimen in figure S4b showing the growth evolution of hexagonal-prism shaped GaN nano- and microcrystals at the inner and outer surfaces in the hollow Aerographite tubular network.

Figure S5 demonstrates the growth evolution of hexagonal prism shaped GaN nano- and microcrystals on the Aerographite hollow tubular network. Growth of hexagonal GaN structures on both inner and outer surfaces can be clearly observed here. The growth of GaN nano- and microstructures also indicates the penetration of the reactants through pores or holes in the AG tubes. The results of our systematic investigation of the morphology of pristine Aerographite and as prepared AG-GaN 3D hybrid network material demonstrate that Aerographite actually consists of highly interconnected tetrapod/multipod-like graphitic microtubes and their walls exhibit holes/or pores (they are sometimes rather tiny). Even having a very thin wall thickness (~ 20 nm), the edges of AG tubes are capable to hold the GaN nanocrystals (Figure S5d).

**Figure S6**


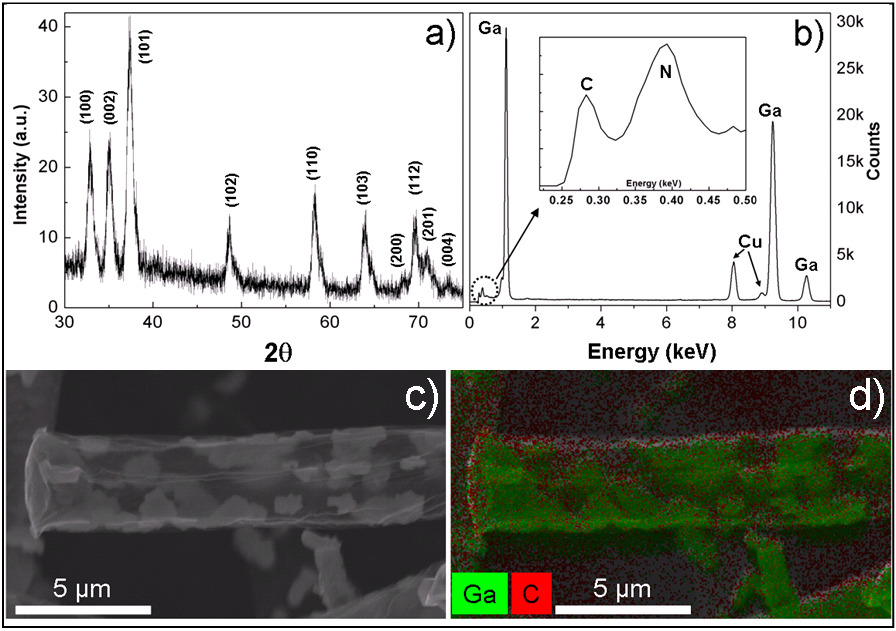


**Figure S6: (**a) XRD pattern of a complete 1 cm^3^ 3D AG-GaN hybrid network. (b) EDX spectrum taken from the GaN nano- and microstructures grown on Aerographite network. (c) SEM image of an individual Aerographite microtube filled with GaN nano- and microstructures. (d) EDX elemental mapping of the GaN nano- and microstructures decorated Aerographite tube (corresponding to c).

The XRD pattern (10 hours scan period) of 3D AG-GaN hybrid network shown in Figure S6(a), reveals that HVPE-grown GaN structures exhibit the (100), (002), (101), (102), (110), (103), (200), (112), (201) and (004) Bragg reflections inherent to GaN and these peaks are in good agreement with that of a randomly oriented GaN powder. Thus the XRD pattern shows that the deposited GaN structures on AG tubes exhibit various crystallographic orientations. There are no visible peaks related to graphitic carbon (expected ~ 2θ= 42°, 44° and 54° ) which is most probably related to the detection limit of the set-up even under long term scan (10 hours). For the chemical composition, EDAX measurement corresponding to 3D AG-GaN hybrid network are shown in Figure S6(b) and S6(c-d) respectively. The spatial distribution of the GaN nano- and microstructures within the AG tubes is homogeneous over large areas of the graphitic network. Figure S6c shows an AG microtube loaded with GaN nano- and microstructures at the inner surface and corresponding EDX elemental map is given in Figure S6d. The elemental map (Figure S6d) clearly confirms the presence of gallium and carbon. The EDX signal of the nitrogen was very weak and therefore in Figure S6d just gallium and carbon are displayed. By employing energy filtered TEM elemental mapping the presence of nitrogen was proved (Figure S6).

**Figure S7**


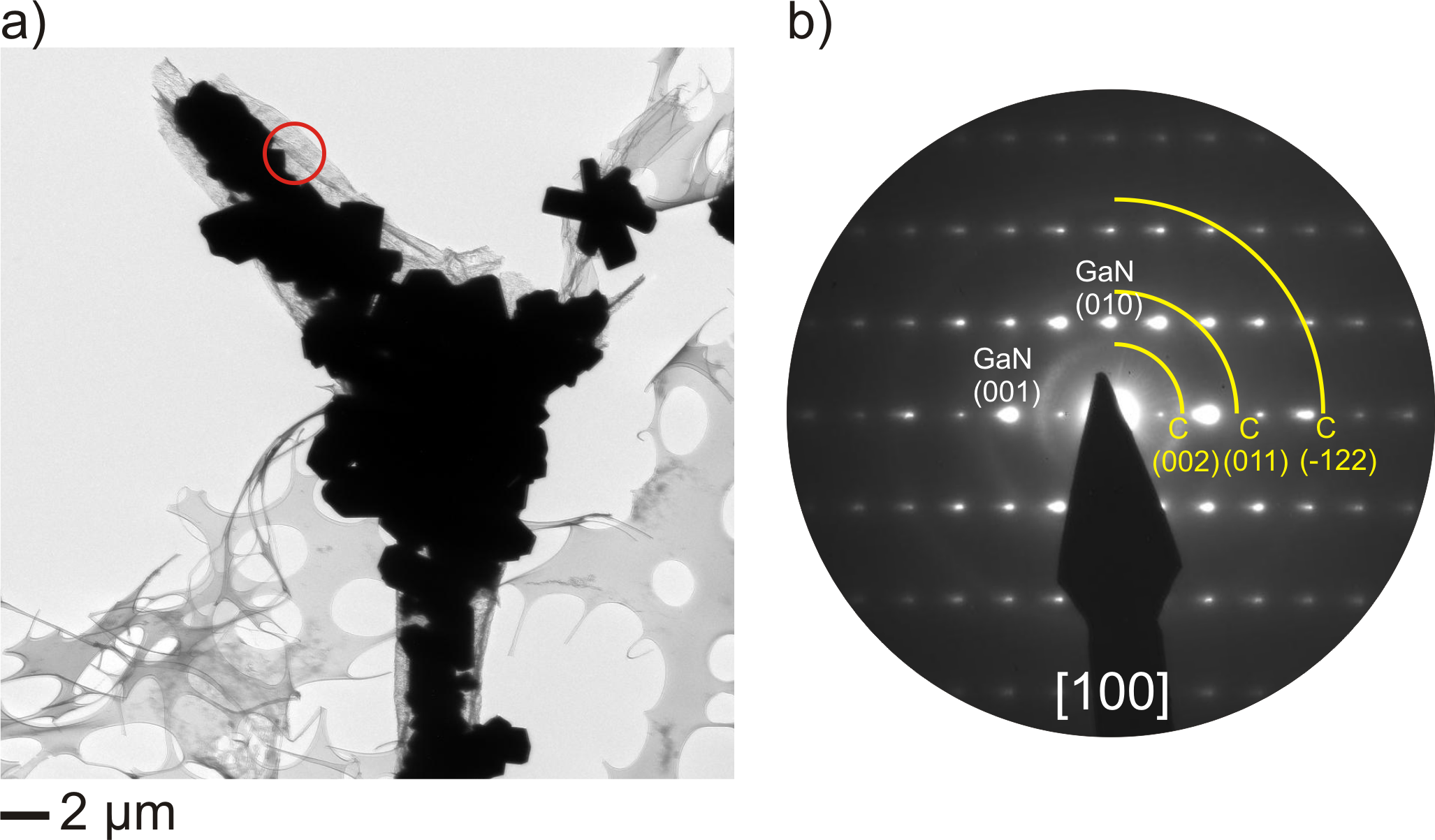


**Figure S7: (**a) TEM micrograph of GaN nano- and microstructures deposited on Aerographite tube (specimen mounted on a TEM grid). (b) SAED pattern from the marked area (circle) in the micrograph (a). The ordered single crystalline diffraction pattern is along GaN [100] zone axis (space group: *P*6_3_*mc*), suggesting a high crystallinity of the deposited GaN structures. The three diffuse rings correspond to (002), (011), (-122) planes of graphite (space group: *P*6_3_/*mmc*).

A further example of a cluster of GaN nano- and microstructures accumulated at the outer and inner surfaces of a broken Aerographite tetrapod is shown in Figure S7a (captured inside the TEM). In Figure S7b the corresponding selected area diffraction pattern is shown which has been recorded at the indicated area in Figure S7a. The diffraction pattern is indexed to be along the [100] zone axis of GaN. In addition to this, three diffused rings are present which originate from the underlying Aerographite and correspond to the (002), (011), (-122) planes of graphite. This is indicative of a poor crystalline quality of Aerographite.
